# Integrated transcriptome analysis identifies APPL1/RPS6KB2/GALK1 as immune-related metastasis factors in breast cancer

**DOI:** 10.1515/med-2023-0732

**Published:** 2023-06-01

**Authors:** Gang Chen, Kun Zhang, Zhi Liang, Song Zhang, Yuanping Dai, Yizi Cong, Guangdong Qiao

**Affiliations:** Department of Breast Surgery, The Affiliated Yantai Yuhuangding Hospital of Qingdao University, Yantai, Shandong 264001, P.R. China; Department of Medical Genetics, Liuzhou Maternal and Child Health Hospital, Liuzhou 545001, P.R. China; Department of Breast Surgery, The Affiliated Yantai Yuhuangding Hospital of Qingdao University, No. 20 Yudong Road, Yantai, Shandong 264001, P.R. China

**Keywords:** breast cancer, immune, metastasis, lymph node, GEO, TCGA

## Abstract

The aim of this study is to investigate the prognostic immune-related factors in breast cancer (BC) metastasis. The gene expression chip GSE159956 was downloaded from the gene expression omnibus database. Differentially expressed genes (DEGs) were selected using GEO2R online tools based on lymph node and metastasis status. The intersected survival-associated DEGs were screened from the Kaplan–Meier curve. Gene ontology (GO) and Kyoto Encyclopedia of Gene and Genome (KEGG) annotation analyses were performed to determine the survival-associated DEGs. Immune-related prognostic factors were screened based on immune infiltration. The screened prognostic factors were verified by the Cancer Genome Atlas (TCGA) database and single-sample gene set enrichment analysis (ssGSEA). As a result, twenty-eight upregulated and three downregulated genes were generated by the survival analysis. The enriched GO and KEGG pathways were mostly correlated with “regulation of cellular amino acid metabolic process,” “proteasome complex,” “endopeptidase activity,” and “proteasome.” Six of 19 (17 upregulated and 2 downregulated) immune-related prognostic factors were verified by the TCGA database. Four immune-related factors were obtained after ssGSEA, and three significant immune-related factors were selected after univariate and multivariate analyses. Based on the risk score receiver operating characteristic, the three immune-related prognosis factors could be potential biomarkers of BC metastasis. In conclusion, APPL1, RPS6KB2, and GALK1 may play a pivotal role as potential biomarkers for prediction of BC metastasis.

## 1 Introduction

Breast cancer (BC) accounts for the majority of new cancer cases and is the second leading cause of cancer-related deaths in female patients in the United States [1]. Approximately 297,790 women will be diagnosed with BC in 2023 [2]. According to molecular characteristics, BC could be divided into at least four subtypes: luminal A, luminal B, human epidermal growth factor receptor 2 positive (HER2+), and triple-negative BC (TNBC) [3]. More than 150,000 BC survivors are living with metastatic disease [4], and BC frequently metastasizes to lymph nodes (LN) [5]. The status of LN metastasis is a prognostic factor in early BC [5] and is highly related to immune infiltration status [6]. Studies indicated that BC with higher immune infiltrating degree may have favorable prognostic outcomes [7,8]. BC metastasis to distant organs is a fatal process and accounts for a majority of BC-related deaths. Once the tumor metastasizes, a surgery is difficult to perform and no effective drugs can be used to cure metastatic BC [9]. The immunotherapy has been generally studied, and triumphantly used in several kinds of metastatic cancers, such as non-small cell lung cancer [10], and melanoma cancer [11]. Because BC has no generally accepted immunogenic therapy targets and immunotherapy in treating BC has not been actively performed [9]. But there is still some immunotherapy clinical research that has been carried in the aggressive BC subtype targeting several immune checkpoints, such as PD-1, CTLA-4, etc. [12]. For the metastasis BC, there is still lack of deep studies.

Here the gene expression omnibus (GEO) dataset GSE159956 was downloaded and categorized into metastasis and non-metastasis groups or LN-positive and -negative groups. Differentially expressed genes (DEGs) were obtained by GEO2R online tools. Overlapping DEGs were analyzed by Kaplan–Meier plotter (KM-plotter). Gene Ontology (GO) and Kyoto Encyclopedia of Genes and Genomes (KEGG) annotation of significant prognosis factor were also performed. Six immune-related prognosis factors were verified by the Cancer Genome Atlas (TCGA) database. Four of the six genes were chosen by single sample gene set enrichment analysis (ssGSEA). Finally, three immune-related prognosis factors were selected by univariate and multivariate Cox analyses.

Although, from the previous reports, we could know that high expression of COLL11A1 was closely related to LN metastasis and involved in the regulation of BC immune infiltration [13], high expression of OSR1 [14] and CXCL14 [15] devote to LN metastasis related death of BC. And CD2 is closely related to immune microenvironment of BC tumors [16]. But combining the LN regulated immune infiltration with distant metastasis was rarely reported.

Hence, LN metastasis, distant metastasis, and immune-related prognostic markers should be identified to accurately predict the potential risks of metastasis and administer therapeutic targets to treat patients with metastatic BC.

## 2 Method

### 2.1 Data collection and processing

The RAN transcriptome series matrix file (GSE159956) was downloaded from the GEO database (https://www.ncbi.nlm.nih.gov/geo/) based on the GPL2567 platform. The patients characteristics and treatment received as previous reported [17,18]. The file consists of 151 LN-positive and 144 LN-negative patients as well as 194 distant metastatic and 101 non-distant metastatic patients. Based on the series matrix file and GPL file, the gene expression matrix file was obtained. Then, the DEGs based on the two groups were analyzed by GEO2R online tools. *P* value ≤0.5 and |log2 fold change (FC)| > 1 were used as the screening standard. Intersected metastasis-related DEGs were obtained by Venn online tools (http://bioinformatics.psb.ugent.be/webtools/Venn/).

### 2.2 GO and KEGG annotations

The GO and KEGG annotations were downloaded from the official websites (http://current.geneontology.org/products/pages/downloads.html, https://www.genome.jp/kegg-bin/get_htext?hsa00001+3101). Data were cleaned into 2 × 2 contingency format using Perl (Version 5.32.1) software, and hypertension formula in R software was used to calculate enrichment values.

### 2.3 Survival analysis

Overlapping DEGs were separated into two groups based on the median expression level, and KM-plotter analysis was conducted to determine prognosis-related metastasis factors using GrandPrism software (Version 5.0). Values at *P* ≤ 0.05 were considered statistically significant.

### 2.4 Immune clustering

Tumor cellularity and the different infiltrating normal cells also called ESTIMATE. The stromal and immune cells that form the major non-tumor constituents of tumor samples promote and facilitate specific signatures related to the infiltration of stromal and immune cells in tumor tissues. The stromal and immune scores usually predict the level of infiltrating stromal and immune cells and these carry the basis for the ESTIMATE score to illustrate tumor purity in tumor tissue [19].

The strength of immune infiltration was categorized into high and low groups to identify immune infiltration affected prognosis factors. Results were verified by ESTIMATE score, immune score, stromal score, and tumor purity. Immune-related prognosis factors were tested by Wilcoxon test, and values at *P* value ≤0.05 were considered significant.

### 2.5 Validation of metastasis prognosis factors by the TCGA database

The TCGA_BRCA dataset and corresponding clinical information were downloaded from the TCGA database by using the TCGA assemble package (Version 2.0) of R software. According to metastasis status, the patients were divided into metastasis and non-metastasis groups. Immune-related prognosis factors were examined by t-test on Grand Prism (Version 5.0) software. Finally, six immune-related prognosis factors were selected.

### 2.6 Identification and confirmation of immune-related prognostic features by the ssGSEA

LASSO regression analysis was performed to confirm the immune-related prognosis factors. High- and low-risk groups were defined by the median of risk score by using the survminer package of R software. Univariate and multivariate Cox regression analyses were conducted, and three prognostic features were selected. Time-dependent receiver operating characteristics (t-ROC) were analyzed to determine the predicting ability of the prognosis factors. From the t-ROC curves, three factors could be used to predict BC metastasis.

### 2.7 Statistical analysis

R software (Version 4.2.2) and GrandPrism were used for statistical analysis. Differences among different risk groups were compared by log-rank test and survival analysis. The *P* value of <0.05 was considered to be statistically significant.

## 3 Results

### 3.1 Analysis of datasets

**Figure 1:**
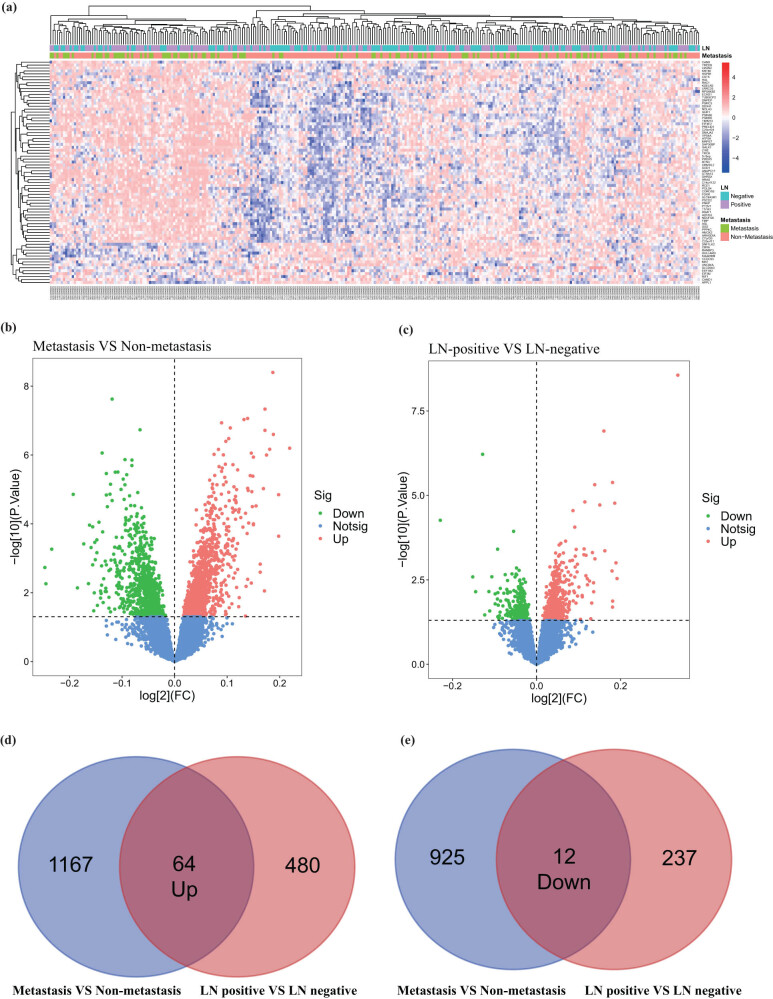
DEGs in metastasis and non-metastasis groups and LN-positive and LN-negative groups. (a) Heatmap of overlapping DEGs based on metastasis status and LN conditions. (b and c) Volcano map of all mRNAs based on metastasis status and LN conditions. (d and e) Overlapping DEGs between metastasis and non-metastasis groups as well as LN-positive and LN-negative groups.

The microarray gene chip GSE159956 was used in this study. The DEGs were analyzed using GEO2R online tools. A total of 1,231 upregulated and 937 downregulated DEGs were found in the distant metastasis group compared with those in the non-distant metastasis group (Figure 1b). About 544 upregulated and 249 downregulated GEGs were obtained under the LN-positive compared with LN-negative conditions (Figure 1c). The overlapped LN metastasis related 64 upregulated and 12 downregulated DEGs were acquired by the Venn diagram (Figure 1a, d, and e).

### 3.2 Survival analysis of DEGs

**Figure 2:**
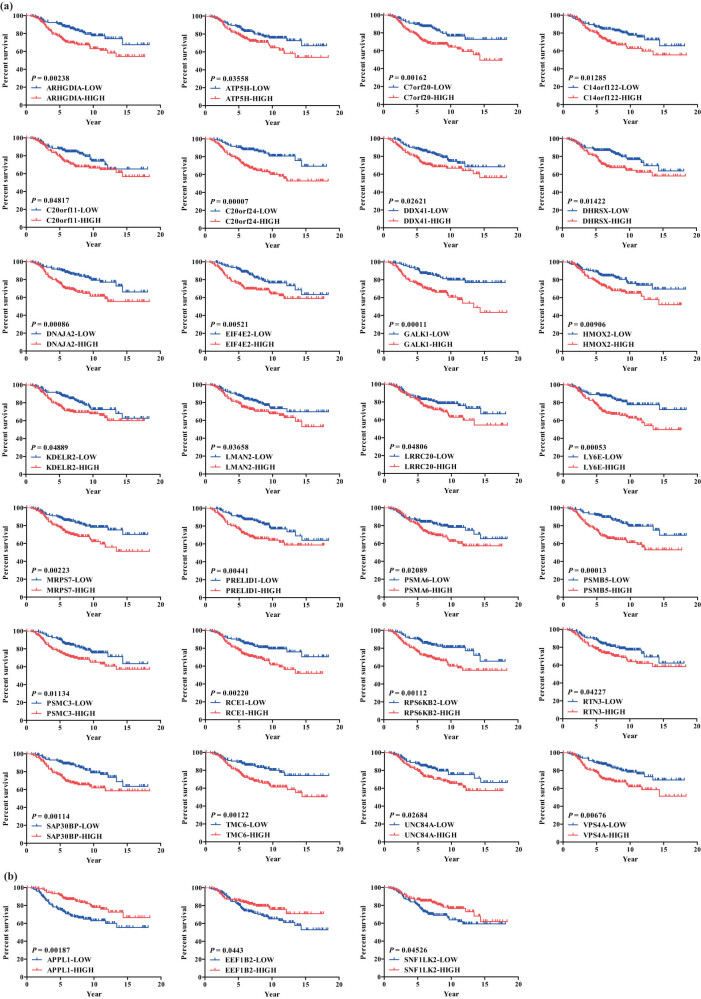
Survival-associated DEGs. (a) Twenty-eight upregulated prognosis-associated DEGs. (b) Three downregulated prognosis-associated DEGs.

In order to obtain the prognosis related DEGs, the prognoses of 72 overlapping DEGs were investigated using KM-plotter. Finally, 28 upregulated and 3 downregulated DEGs were found to be involved in the prognosis (Figure 2a and b).

### 3.3 GO and KEGG annotations

**Figure 3:**
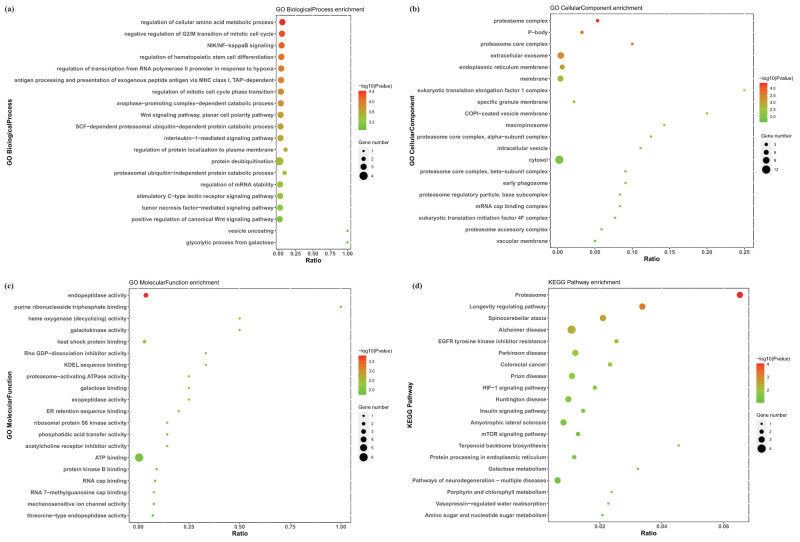
GO and KEGG enrichment analyses of survival-associated DEGs. (a–c) GO analysis of survival-associated DEGs. (d) KEGG analysis of survival-associated DEGs.

For the survey of the potential function and pathway of 31 prognostic DEGs, GO and KEGG annotations were performed. Prognostic DEGs were mostly enriched in the “regulation of cellular amino acid metabolic process,” “proteasome complex,” “endopeptidase activity” in biological process and cellular component, and molecular function segments (Figure 3a–c). The “Proteasome” KEGG pathway was mostly enriched (Figure 3d). From the KEGG and GO annotation we can know that proteasome related amino acid metabolic process may be mostly involved in the distant and LN metastasis process.

### 3.4 Immune clustering and verification

**Figure 4:**
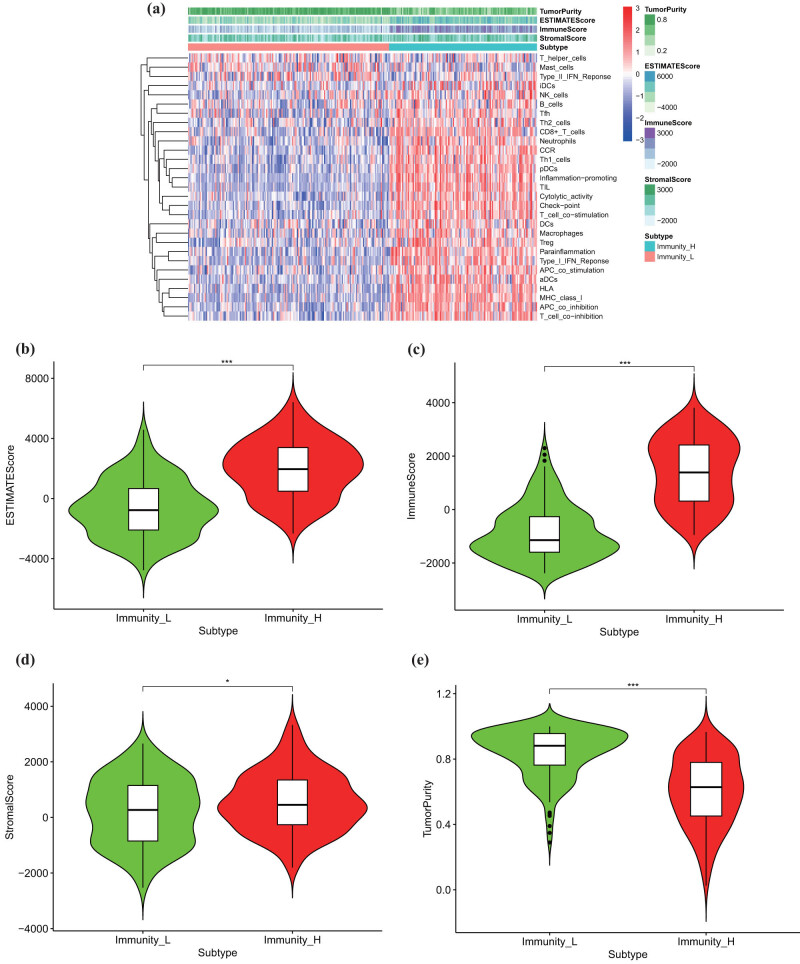
Construction and validation of BC immune infiltration clustering. (a) Enrichment levels of 29 immune-related cells and types in the high and low immune infiltration groups. The ESTIMATE Score, Stromal Score, Immune Score, and Tumor Purity of every patient combined with the clustering information. (b–e) Differences in ESTIMATE Score, Stromal Score, Immune Score, and Tumor Purity, respectively, between two clusters.

**Figure 5:**
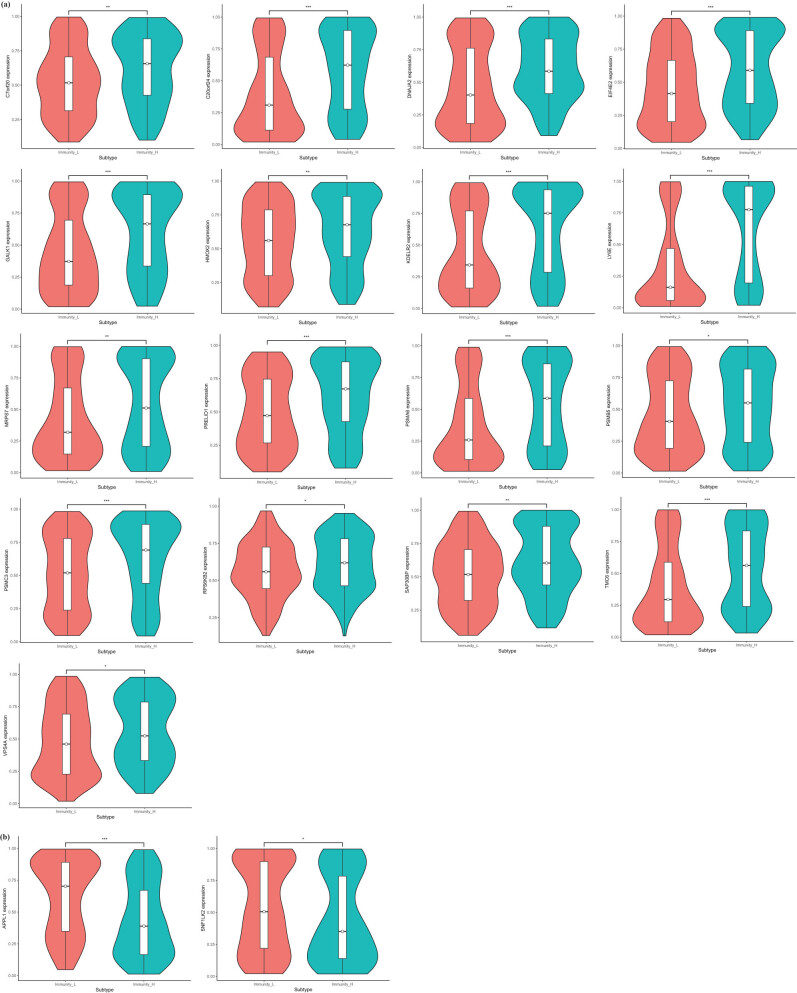
Immune infiltration-associated DEGs. (a) Seventeen upregulated immune infiltration-associated DEGs. (b) Two downregulated immune infiltration-associated DEGs.

The samples were separated into high and low immune infiltration clusters and verified using ESTIMATE score, immune score, stromal score, and tumor purity (Figure 4a–e). Immune-related prognosis factors were evaluated by Wilcoxon test, and 17 upregulated and 2 downregulated factors were significantly influenced in the high and low immune infiltration clusters (Figure 5a and b).

### 3.5 Six metastasis prognosis factors were picked out by the TCGA database

**Figure 6:**
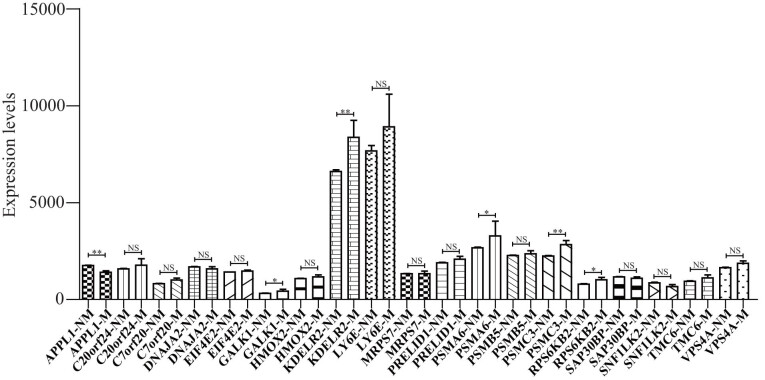
Expression validation of 19 immune infiltration-associated DEGs by using TCGA datasets. Five upregulated and one downregulated immune infiltration-associated DEGs were verified in metastasis (M) and non-metastasis (NM) groups by using the TCGA database.

According to the distant metastasis status, the TCGA_BRCA dataset expression files were divided into two parts. The abovementioned 17 upregulated and 2 downregulated immune prognostic features were compared, and 5 upregulated and 1 downregulated features were significantly different in the non-distant metastasis and distant metastasis groups (Figure 6).

### 3.6 Three immune-associated prognostic features were recognized by the ssGSEA

**Figure 7:**
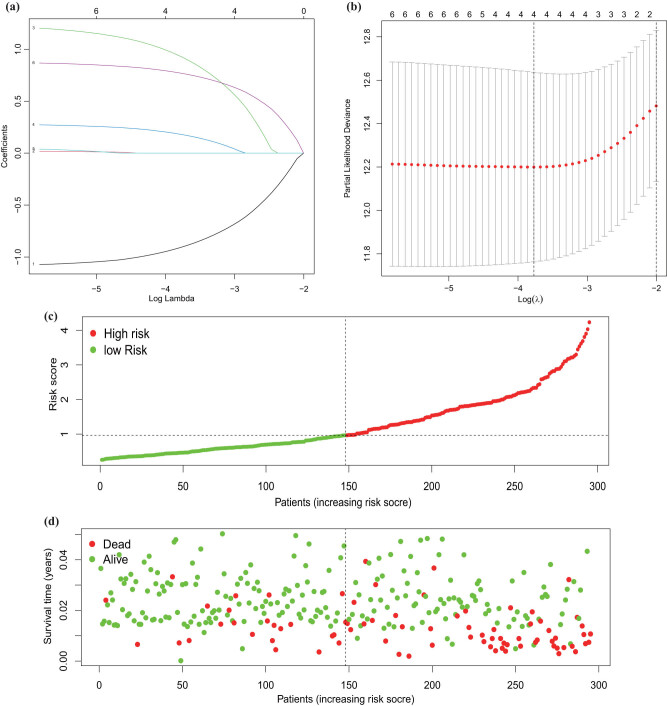
Verification of immune-related prognostic genes by LASSO regression. (a) The LASSO Cox analysis identified four genes associated with prognosis. (b) The optimal values of the penalty parameter were defined by 1,000-round cross validation. (c) The risk curve of every sample was arranged by risk score. (d) The scatter plot of BC samples indicating survival.

**Figure 8:**
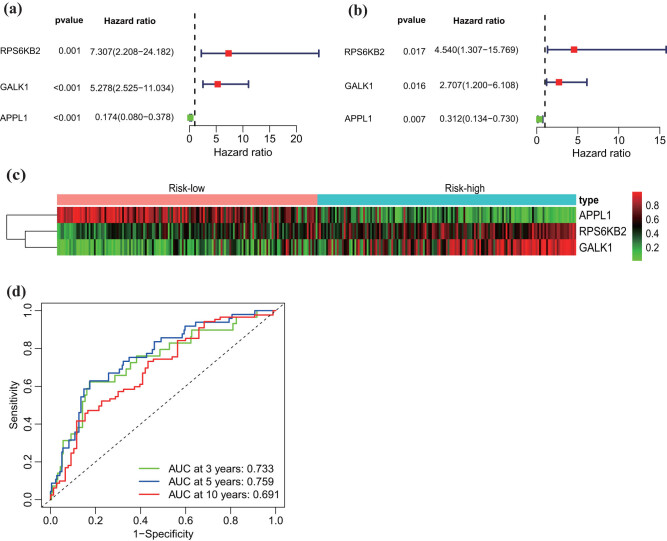
Evaluation of independent prognostic value. (a) Univariate and multivariate analyses and (b) Cox regression, where three genes were selected. (c) The heatmap of three genes based on risk score. (d) AUC of the three genes based on the ROC curve.

LASSO regression was carried out, and four prognosis factors were selected (Figure 7a and b). High- and low-risk groups were separated based on the median of risk score. The survival status of the patients is shown in Figure 7c and d. Consequently, univariate and multivariate Cox regression analyses showed that two upregulated (*RPS6KB2* and *GALK1*) and one downregulated (*APPL1*) prognosis factors were significant. The results of the univariate and multivariate analyses are shown in Figure 8a and b, and the heatmap is shown in Figure 8c. Furthermore, t-ROC was produced, and the areas under the curve (AUC) were 0.733, 0.759, and 0.691 in 3, 5, and 10 years, respectively (Figure 8d). This finding indicated that the three genes influenced by the LN status and immune infiltration could be used as prognostic factors to predict BC distant metastasis.

## 4 Discussion

BC is the leading cause of cancer-associated deaths among women worldwide [20]. Because of breast screening, most of the patients are diagnosed at early stage, which has a 5 year survival rate and can be as high as 100% [21]. Although most patients with early BC can be cured, a considerable number of patients, 20–30%, will still develop local recurrence or distant metastasis within 2 years of diagnosis of the primary tumor [22,23]. And causes of high incidence rate for BC patients [23]. BC cells are usually spread by lymphatic or hematogenous mode, and LN is often the first site of metastasis; LN-positive status can greatly increase the risk of the distant metastasis of BC [24,25].

Here the gene expression file GSE159956 was downloaded from the GEO database. A total of 295 patients were categorized into two groups based on metastasis status and LN status. LN-affected by metastasis genes was selected using GEO2R online tools. The overlapping 31 prognosis-related DEGs were selected using KM-plotter. GO and KEGG annotations were performed to determine the potential function of prognostic features, and the results showed that amino acid metabolic related pathway may influence the BC distant metastasis. For the known immune related prognostic factors, patients with high and low immune infiltration rates were clustered and verified by ESTIMATE, stromal scores, and tumor purity. Nineteen immune-associated prognosis factors were obtained by Wilcoxon test based on the high and low immune infiltrating groups. In addition, 6 of the 19 factors were confirmed by the TGCA_BRCA dataset. After LASSO regression and univariate and multivariate analyses, one downregulated (*APPL1*) and two upregulated (*RPS6KB2* and *GALK1*) immune-related metastatic factors were selected. Finally, from the t-ROC we could know that the three factors could be used to predict BC metastasis. The detailed information about the 3 prognostic factors is presented as follows.

APPL was originally found as an AKT2 binding protein in a yeast two-hybrid screening system [26] and is named after its unique structure, an adaptor protein containing pleckstrin homology domain, phosphotyrosine binding domain, and leucine zipper motif [27]. *APPL1* has implicated roles in insulin sensitivity and regulating insulin signaling pathways [28,29]. In addition, it affects cell functions, such as cell growth, migration, apoptosis, prognosis, endosomal trafficking, etc., by regulating some signaling events [27,30,31]. The expression levels of APPL1 was not only downregulated in kidney renal clear cell carcinoma tissues, and closely relate with Treg infiltration and immune checkpoints, but also inhibits Caki-1 cell migrations and growths [32]. Yet, APPL1 was highly expressed in the prostate cancer tissues [33]. Whereas, the functions of APPL1 on the BC metastasis are still unclear. In the present study, we found that low *APPL1* expression could be used as a potential BC metastasis biomarker.

*RPS6KB2*, also known as *S6K2*, is the unheeded member of the S6K family [34] and shares nearly 80% of the amino acid sequence with the studied homolog *S6K1*. *RPS6KB2* undertakes a downstream effective apparatus of PI3K/AKT/mTOR and RAS/RAF/MEK/ERK pathways [34]. Therefore, *RPS6KB2* is usually linked to cell proliferation and prognosis, such as in BC and prostate cancer [35,36]. High *RPS6KB2* expression is correlated with chemotherapy resistance and prognosis of BC patients [37], indicating its potential role in cancer treatment. *RPS6KB2* is also highly expressed in about 5% of patients with gastric carcinoma [38]; this high expression is associated with decreased overall survival rates of patients with the late-stage disease [34]. Hence, *RPS6KB2* may be a BC metastasis indicator.

Galactokinase (*GALK1*) plays an important role in the first stage of catalysis metabolism of galactose and the conversion of galactose into galactose-1-phosphate at the consumption of ATP [39,40]. In addition, *GALK1* could be a new therapeutic target for liver cancer treatment [41]. Inhibiting GALK1 could reduce the proliferation rate of HepG2 cells [42]. *GALK1* in BC has been rarely reported. Here we used integrated bioinformatics methods and found that GALK1 could be a biomarker for predicting BC metastasis.

## 5 Conclusion

We identified three BC distant metastasis-related genes that were found to be significantly associated with prognosis. Combining with LN status, the three genes could be used to predict BC distant metastasis. However, further validations in clinical experiments are needed. These findings provide an approach for predicting BC distant metastasis and potential therapeutic targets for BC treatment.
